# New gait performance indices and cognitive functions: a longitudinal pre–post follow-up after rehabilitation in people with Parkinson’s disease

**DOI:** 10.3389/fnhum.2026.1791254

**Published:** 2026-05-07

**Authors:** Elena Sofia Cocco, Raimondo Stefano Maria Torcisi, Carrie-Louise Thouant, Mohamed El Arayshi, Luca Pietrosanti, Carlotta Maria Manzia, Annalisa Gison, Paola Romano, Leonardo Buscarini, Francesco Infarinato, Marco Franceschini, Cristiano Maria Verrelli, Sanaz Pournajaf

**Affiliations:** 1 Department of Neuromotor Rehabilitation and Rehabilitation Robotics, IRCCS San Raffaele Roma, Rome, Italy; 2 Rehabilitation Bioengineering Laboratory, IRCCS San Raffaele Roma, Rome, Italy; 3 Electronic Engineering Department, University of Rome Tor Vergata, Rome, Italy; 4 Department of Neuroscience and Neurorehabilitation, IRCCS San Raffaele Roma, Rome, Italy; 5 Department of Mental and Physical Health and Preventive Medicine, University of Campania “Luigi Vanvitelli”, Naples, Italy

**Keywords:** Fibonacci sequence, gait analysis, lower limb, Parkinson’s disease, rehabilitation, self-similarity, wearable inertial sensors

## Abstract

**Introduction:**

Gait impairment and motor–cognitive interaction are key features of Parkinson’s disease (PD) and critically affect functional autonomy and fall risk. Wearable inertial sensors allow for objective assessment of gait performance, while the Ф-bonacci gait number has been shown to provide a composite gait performance measure integrating self-similarity, symmetry, and double-support consistency. Building on previous cross-sectional findings, this observational pilot study represents a longitudinal follow-up that extends the application of the Ф-bonacci index framework towards the monitoring of walking performance progression over time. Indeed, this pilot study investigated changes in gait performance and motor–cognitive interaction in people with PD, using conventional clinical scales and the Ф-bonacci gait number.

**Methods:**

Nineteen individuals with PD - carrying out conventional outpatient multidisciplinary rehabilitation - underwent clinical and instrumented gait assessments at baseline (T0) and one-month later (T1). Gait data were collected during the 6-Minute Walk Test using wearable inertial sensors. The changes in spatiotemporal parameters, the Ф-bonacci gait number and its components, and the dual-task performance (Timed Up and Go Dual Task, TUG-DT) were analyzed, together with associations between gait harmonicity, clinical motor improvement, and dual-task performance.

**Results and discussion:**

Participants at T1 showed significant improvements in functional mobility and spatiotemporal gait parameters, along with a reduction in the Ф-bonacci gait number, mainly driven by enhanced gait self-similarity and double-support consistency. Such improvements in gait harmonicity were significantly associated with both clinical motor improvement and enhanced dual-task performance, while global cognitive scores remained stable. These findings support the claim that Ф-bonacci gait number is a longitudinal marker sensitive to harmonic gait performance in PD and suggest a condition-dependent motor-cognitive coupling in the presence of dual-task demands.

## 1 Introduction

Parkinson’s disease (PD) is a common neurodegenerative syndrome characterized by a progressive development of symptoms (Lei et al., 2019), affecting approximately 10 million people worldwide, with an estimated prevalence of about 2% among individuals over the age of 80 years (Gualerzi et al., 2024). Although the precise etiology of PD remains unknown, current research focuses on a combination of genetic and environmental factors (Hou et al., 2019; Schapira, 1997). The clinical manifestations of PD include both motor symptoms—such as tremor, bradykinesia, and rigidity—and neuropsychiatric symptoms, including depression, anxiety, memory deficits, and anosmia (Leng et al., 2020). Among motor symptoms, physical exercise has been demonstrated to play a pivotal role in their management, contributing significantly to improvements in quality of life (QoL) (Armstrong and Okun, 2020). Exercise regimens should be tailored to the individual motor profile and conducted in a safe environment to minimize the risk of adverse events (Kalbe et al., 2023). In accordance with the International Classification of Functioning, Disability and Health (ICF), PD affects multiple domains, including body functions, activities, and participation (Stucki et al., 2002). Therefore, physical exercise has emerged as an essential component in the treatment of people with Parkinson’s disease (pwPD) (Krause et al., 2022). Within this framework, the growing adaptation of multidisciplinary and multifactorial rehabilitative treatment in a rehabilitation program delivered on a non-residential basis is closely supported by clinical assessment scales such as the Hoehn and Yahr Scale (H&Y), the Unified Parkinson’s Disease Rating Scale (UPDRS), the Timed Up and Go Test (TUG), and the Six-Minute Walk Test (6MWT), which are essential for monitoring motor and cognitive symptoms (Goffredo et al., 2024). Furthermore, gait can be assessed not only qualitatively but also quantitatively: the gold standard for instrumented gait assessment remains the marker-based motion capture system, despite its complexity and high cost (Bailo et al., 2024). In contrast, Inertial Measurement Units (IMUs) offer a sensitive and objective alternative that is cost-effective, user-friendly, eco-friendly, and non-invasive (Tramontano et al., 2024; Ghattas and Jarvis, 2024; Cappozzo et al., 2005; Romano et al., 2021; El Arayshi et al., 2022; Iosa et al., 2016; Verrelli et al., 2021; Saggio et al., 2021). Now, in pwPD, the fluid and rhythmically consistent flow of movement is often diminished, and gait self-similarity is typically disrupted (Iosa et al., 2016). Recently, the Ф-bonacci gait number and the concept of Harmonic Gait Variability (HGV) have been introduced, enabling the quantitative measurement of deviation from a harmonic reference gait model (Verrelli et al., 2021). Traditional gait measures (speed, stride length, cadence) quantify magnitude of motor output. The Φ-bonacci index instead captures the internal temporal organization of gait cycles, integrating harmonicity, inter-limb coordination, and double-support structure into a single descriptor. Therefore, it detects subtle reorganizational changes not reflected by isolated spatiotemporal metrics and supports clinicians in evaluating gait cycle self-similarity (harmonicity), asymmetry, double-support consistency, providing valuable data to inform rehabilitation strategies (Verrelli et al., 2021). In this respect, cyclic human movements, such as gait cycles were found, with their given internal sub-phases, to be characterized, from a temporal point of view, by the existence of coordinatively mechanized and self-similar harmonic fractal patterns. Such harmonic patterns are implicitly defined by the golden ratio, when it occurs as the ratio of the sub-phases durations, symmetrized in time so as to compose a generalized Fibonacci sequence. The lowest amount of information for the movement temporal design makes one sub-phase duration of the movement temporally generate an entire sequence of sub-phase durations of the same movement. In particular, generalized Fibonacci sequences are sequences of numbers that are generated by the first two elements (called seeds) via the following generation rule: from the third number, every element of the sequence is the sum of the previous two. As far as symmetric walking is concerned—where the stance duration (third element of the sequence) is the sum of the double support and swing durations (first and second elements of the sequence, respectively) and, in turn, the duration of the entire gait cycle (fourth element of the sequence) is the sum of the stance and swing durations—generalized Fibonacci sequences were discovered to provide a walking gait characterization in terms of a completely automatized execution. Furthermore, the golden ratio was found to characterize hidden self-similar patterns: all the ratios between two consecutive elements of the sequence (namely, the durations of the sub-phases of the movement) are surprisingly equal. The temporal partition of a movement into phases that maintain the same proportion in their ratios appears to be an optimization strategy for addressing the motor equivalence problem associated with the coordinated control of numerous degrees of freedom.

This observational pilot study aims to complement our previous paper (Cocco et al., 2025). It provides an analysis of gait patterns in pwPD by assessing biomechanical data and comparing gait harmonicity—quantified via the Ф-bonacci number—before and after a conventional multidisciplinary rehabilitative intervention delivered in an outpatient setting. Additionally, the study seeks to explore correlations between motor coordination, measured using validated clinical assessment tools, and temporal structure of gait.

In this paper, which can be thus viewed as a sequel to the previous one, we mainly apply the same general methodology therein to new data covering the following additional scenario: the observational study required that the participants were enrolled and assessed at baseline, then completed a one-month multidisciplinary rehabilitation program and subsequently were assessed (post-rehabilitation) with the same assessments as at baseline. As a new contribution, we show how it is possible to obtain up-to-date and meaningful information for the purpose of personalizing a rehabilitation program, promptly responding to the needs of the pwPD.

In PD, gait automaticity is reduced due to basal ganglia dysfunction, leading to increased reliance on cortical and executive control mechanisms. Under dual-task conditions, motor and cognitive systems compete for shared attentional resources. Therefore, gait harmonicity may represent a functional marker of the efficiency of motor–cognitive integration. Accordingly, this study examined the longitudinal association between motor performance and dual-task outcomes in pwPD undergoing standard outpatient rehabilitation. In the presence of stable global cognitive scores, we investigated whether changes in motor-related parameters, including gait harmonicity, were accompanied by corresponding changes in dual-task performance. The aim is to explore the extent to which motor and motor–cognitive domains evolve in parallel over time and to evaluate the longitudinal sensitivity of the Φ-bonacci gait number in relation to clinical motor changes and dual-task performance in pwPD.

## 2 Materials and methods

### 2.1 Retrospect

The methodological contribution of this work, compared to what was proposed in (Cocco et al., 2025), is of an incremental nature: the same framework previously described has been maintained, but some targeted extensions have been introduced that allow us to delve deeper into the evolutionary dynamics observed over time.

For the sake of clarity, the Φ-bonacci gait number in (Cocco et al., 2025) is reported. This index is defined as a composite gait harmonicity descriptor integrating the three fundamental components introduced in the original formulation—self-similarity (*A*₁) (Equation 1), swing-phase symmetry (*A*₂) (Equation 2), and double-support (DS) consistency (*A*₃) (Equation 3)—whose weighted and normalized combination yields the Ф-bonacci gait number, a unified measure of harmonicity, coordination, and temporal organization of walking (Equation 4).


$$A_{1}=\sqrt{{\left(\frac{{\mathrm{SW}}_{l}}{{\mathrm{DS}}_{r}}-\phi \right)}_{n}^{2}+{\left(\frac{{\mathrm{SW}}_{r}}{{\mathrm{DS}}_{l}}-\phi \right)}_{n}^{2}+\mu^{\mathrm{adj}}{\left(\frac{{\mathrm{SW}}_{r}^{\mathrm{adj}}}{{\mathrm{DS}}_{l}^{\mathrm{adj}}}-\phi \right)}_{n}^{2}}$$ (1)

$$A_{2}=\lambda \sqrt{{\left(\frac{{\mathrm{SW}}_{r}}{{\mathrm{SW}}_{l}}-1\right)}_{n}^{2}+\lambda^{\mathrm{adj}}{\left(\frac{{\mathrm{SW}}_{r}^{\mathrm{adj}}}{{\mathrm{SW}}_{r}}-1\right)}_{n}^{2}}$$ (2)

$$A_{3}=\delta \sqrt{{\left(\frac{{\mathrm{DS}}_{x}}{{\mathrm{DS}}_{y}}-1\right)}_{n}^{2}}$$ (3)

Equation 1 quantifies the *self-similarity* of gait by comparing the relative proportions of stance (ST), swing (SW), and double support (DS) phases between a given gait cycle (GC) and its adjoint (i.e., the corresponding cycle of the contralateral limb). When the temporal organization of subphases maintains the same internal ratios across successive cycles, the deviations vanish and *A*₁ approaches zero, reflecting a highly harmonic and recursively structured gait pattern. Equation 2 captures *swing-phase symmetry* by evaluating the difference between right and left SW durations, again involving also the adjoint cycle. In an ideally symmetrical locomotor pattern—where both limbs contribute equally to the cyclic alternation—*A*₂ tends toward zero, indicating optimal bilateral coordination. Equation 3 describes double-support consistency, which measures the stability and time invariance of the DS sub-interval across cycles. Since the DS phase reflects inter-limb transfer of weight and dynamic balance, higher variability increases *A*₃, whereas a consistent and evenly timed DS phase leads *A*₃ to 0, denoting stable locomotor transitions. In technical terms, the Φ-bonacci gait number turns out to constitute the most natural generalization, to the non-symmetric and recursive walking case, of the corresponding gait ratio |SW/DS − *φ*| defined in (Iosa et al., 2016; Iosa et al., 2013) for symmetric walking, while it simply incorporates a weighted modification of the index = |SW|/SW in (Blazkiewicz et al., 2014), evaluated at both the gait and the adjoint gait.


$$\begin{array}{l} \phi_{b}=\sqrt{{\left(\frac{{\mathrm{SW}}_{l}}{{\mathrm{DS}}_{r}}-\phi \right)}_{n}^{2}+{\left(\frac{{\mathrm{SW}}_{r}}{{\mathrm{DS}}_{l}}-\phi \right)}_{n}^{2}+\mu^{\mathrm{adj}}{\left(\frac{{\mathrm{SW}}_{r}^{\mathrm{adj}}}{{\mathrm{DS}}_{l}^{\mathrm{adj}}}-\phi \right)}_{n}^{2}}+\\ \lambda \sqrt{{\left(\frac{{\mathrm{SW}}_{r}}{{\mathrm{SW}}_{l}}-1\right)}_{n}^{2}+\lambda^{\mathrm{adj}}{\left(\frac{{\mathrm{SW}}_{r}^{\mathrm{adj}}}{{\mathrm{SW}}_{r}}-1\right)}_{n}^{2}}+\delta \sqrt{{\left(\frac{{\mathrm{DS}}_{x}}{{\mathrm{DS}}_{y}}-1\right)}_{n}^{2}} \end{array}$$ (4)

### 2.2 Study design

This observational pilot study aimed to evaluate the longitudinal sensitivity of the Φ-bonacci gait number in relation to clinical motor changes and dual-task performance in pwPD undergoing conventional outpatient multidisciplinary rehabilitation. This study adheres to the Declaration of Helsinki and was approved by the local ethics committee (PR no. 21/30 of December 2021).

### 2.3 Participants

Twenty-two people with PD, recruited from the Day Hospital Department of the IRCCS San Raffaele (Rome, Italy), were included in the study. All recruited individuals signed informed consent and met the inclusion criteria stated in the study protocol. Inclusion criteria included: age between 30 and 80 years; diagnosis of Parkinson’s disease according to the Movement Disorder Society (MDS); H&Y (Goetz et al., 2004) scale score between 2 and 3 in the “on” phase; Montreal Cognitive Assessment (MoCA) screening test score ≥22 (Cocco et al., 2025); MDS-UPDRS Part IV score ≤2 in both items (duration and disability) related to dyskinesias; stabilized pharmacological treatment; ability to understand and sign the informed consent form; and ability to comply with the procedures set out in the study protocol. People with neurological conditions concomitant with Parkinson’s disease, psychiatric complications, or personality disorders; musculoskeletal disorders that could impair movement; severe language deficits that prevented comprehension and compliance with experimental procedures; or failure to provide informed consent were excluded from the study. Although 22 participants were recruited, data from only 19 participants were analyzed. Three participants were excluded from the instrumental gait analysis due to insufficient signal quality during the 6MWT (*n* = 1 technical artifact; *n* = 2 premature interruption of the test). Fifteen healthy participants (age between 35 and 85 years old, absence of pathologies affecting the lower limb function and cognitive and/or severe visual deficit) were included exclusively to provide normative reference values for the Φ-bonacci index, based on previously collected data (Cocco et al., 2025).

### 2.4 Demographic and clinical assessments

The following demographic data were recorded for each study participant: age, sex, weight, height, date of disease diagnosis, medication use, and time of last dose of medication before the walking test. The overall degree of disease-related disability was assessed using several clinical scales. Specifically, the following were administered: MDS-UPDRS total and subtotals (scores from Parts I, II, III, and IV); H&Y; MoCA; Mini-BESTest; 6MWT; TUG; TUG-DT; 10MWT; NFOG-Q; ABC.

All measurements (at T0 and T1) were acquired in the “ON” phase, i.e., 60 ± 20 min after oral intake of the usual dose of Levodopa, and always in the morning to minimize variability. All participants followed stabilized pharmacological treatment. The evaluation was performed by highly specialized clinical staff at the IRCCS San Raffaele in Rome.

### 2.5 Experimental setup

**Figure 1:**
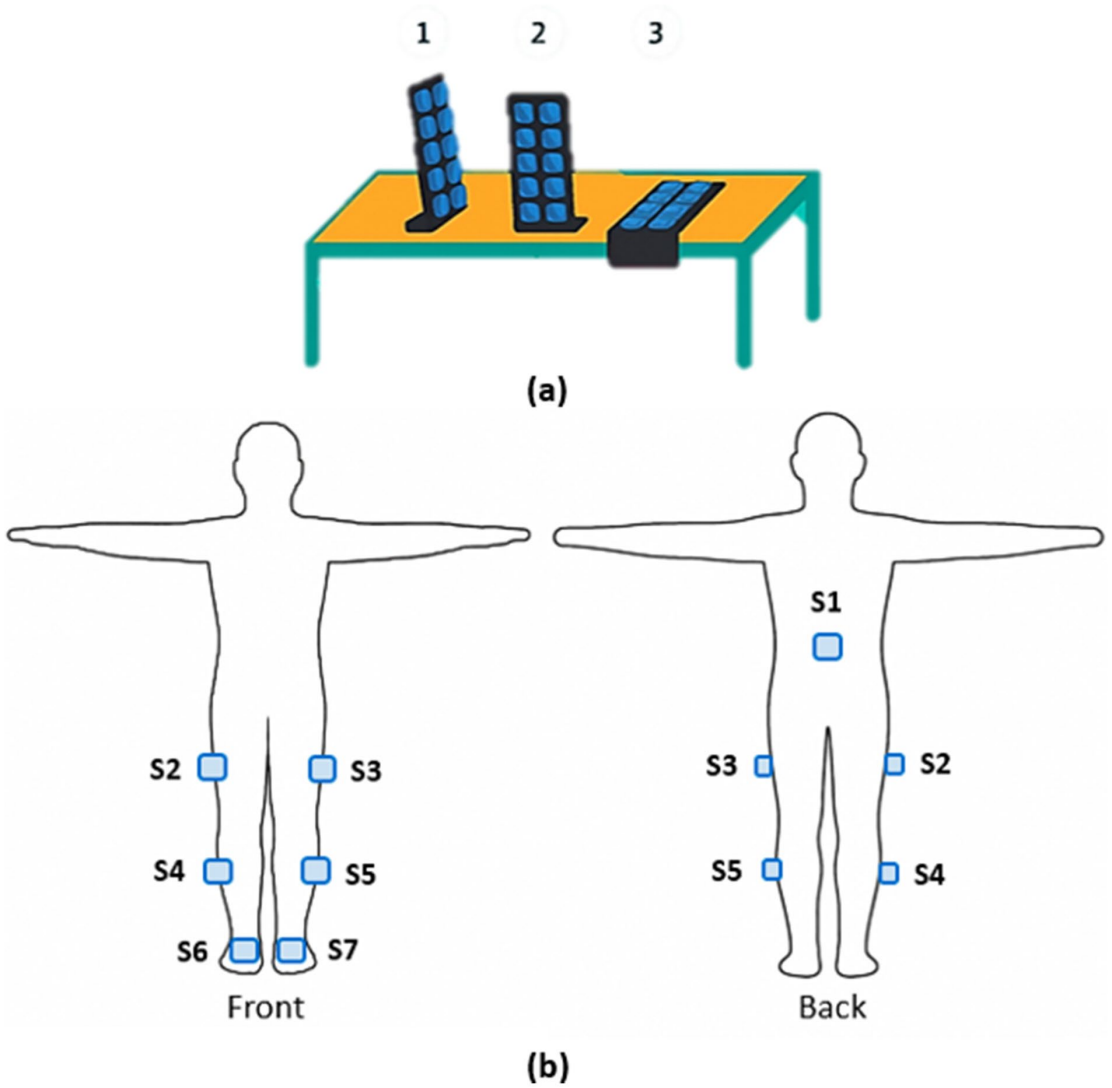
Experimental setup. **(a)** First-step calibration procedure. **(b)** Second-step calibration procedure (“T-pose”) and IMUs’ positioning.

The study was conducted at IRCCS San Raffaele in Rome (Italy), which is equipped with the Movit System G1 by Captiks for Motion Capture and Motion Analysis (Captiks s.r.l., Rome, Italy). Each participant performed the 6MWT along a 15-meter corridor, while wearing the Movit system, which consisted of seven inertial measurement units (IMUs). Each Movit sensor (dimensions: 48 × 39 × 18 mm; weight: 40 g) integrates a triaxial accelerometer and a triaxial gyroscope, providing spatial orientation via a six degrees-of-freedom (6 DoF) quaternion. Based on a patented two-step calibration procedure, the orientation of each sensor is mapped to the corresponding body joint, enabling the reconstruction of joint kinematics and anatomical angles. The first calibration step involves capturing three positions through two 90° rotations using a calibration base, which establishes a unique reference system. The second step consists of acquiring the “T-pose” (standing upright with upper limbs extended horizontally) to align (Functional Alignment-FA) the Movit system with the body coordinate system (Figure 1).

The seven sensors were placed at (Figure 1):

- Trunk: at the level of the L5 lumbar vertebra.
- Thighs: mid-lateral aspect of the right and left femur.
- Shanks: mid-lateral aspect of the right and left fibula.
- Feet: dorsal surface of the right and left foot.

The sensors were secured to the body using Velcro straps, applied over the participants’ clothing. The garments were chosen to ensure stability during the walking test.

The MOVIT system has been validated against a video-based reference system (Saggio et al., 2021) (Vicon, Oxford Metrics), demonstrating high accuracy and repeatability, with root mean square errors (RMSE) of joint angles below 3.5°.

### 2.6 Rehabilitation program

All participants enrolled in the study underwent a 30-days outpatient rehabilitation program, delivered three times per week with a total duration of approximately three hours per session. The intervention was multidisciplinary. It consisted of an integrated set of therapeutic activities, including i) physiotherapy, aimed at improving motor function and postural stability; ii) occupational therapy, focused on enhancing performance in activities of daily living; iii) speech therapy, targeting communication and respiratory support; and iv) psychological interventions, designed to strengthen cognitive abilities and address emotional and behavioral aspects associated with the condition. Each session comprised structured physiotherapy focused on gait training—particularly step rhythm, length, and amplitude; static and dynamic balance exercises; and postural control interventions aimed at enhancing trunk extension and openness. The program incorporated progressive difficulty and task-specific practice. Exercise intensity was individually tailored according to the patient’s clinical condition and tolerance. Occupational therapy targeted the execution of functional tasks, while speech therapy included respiratory and phonatory exercises. This multimodal approach reflects standard clinical practice and ensures a comprehensive and coordinated management of patients’ rehabilitative needs.

### 2.7 Signal processing and statistical analysis

The data acquisition and processing procedures were identical to those previously described in Cocco et al. (2025). Briefly, gait data were recorded using the Captiks Motion Capture system (Rome, Italy), and temporal events (heel strike and toe-off for each foot) were automatically extracted using the Motion Analyzer software. The Ф-bonacci gait number was computed according to the previously validated algorithm, integrating gait self-similarity, symmetry, and double support consistency within a single composite metric (Cocco et al., 2025; Verrelli et al., 2025; Fahn et al., 1987).

In the present study, this methodology was applied to both baseline (T0) and post-treatment (T1) sessions, enabling a longitudinal comparison of gait harmonicity and its relationship with functional improvements. Data from all participants were analyzed to assess changes between T0 and T1 for the Ф-bonacci gait number and the TUG-DT.

Primary outcomes were the *Φ*-bonacci gait number and TUG-DT. Secondary outcomes included functional mobility measures (TUG and 6MWT). Spatiotemporal gait parameters and Φ-bonacci components (A1, A2, A3, GC) were considered exploratory. Effect sizes were calculated for primary outcomes and key secondary functional measures using Cohen’s *d* based on pooled standard deviations derived from reported group means and standard deviations. Ninety-five percent confidence intervals (95% CI) were estimated using standard error approximation. Because individual-level paired variance estimates were not available for table-based computation, pooled effect sizes were reported. Other secondary and exploratory variables were interpreted based on non-parametric comparisons only.

For each participant, individual trajectories between T0 and T1 were displayed using paired line graphs, in normalized form. Because the variables of interest (Ф-bonacci gait number and TUG-DT) have different units and dynamic ranges, individual trajectories between T0 and T1 were normalized using a baseline-ratio method. Specifically, for each subject *i* and each variable X, the normalized value at T1 was computed as (Equation 5):


$$X_{\mathrm{norm}}(i,T_{1})=\frac{X(i,T_{1})}{X(i,T_{0})}$$ (5)

while the baseline was set to 1. This approach produces dimensionless quantities that preserve within-subject changes while allowing direct visual comparison of trajectories across metrics. Values >1 indicate relative increases from baseline, whereas values <1 reflect decreases. The direction of improvement was defined as a decrease in TUG-DT values and Ф-bonacci gait number (i.e., lower post-intervention scores indicating better performance). Correlation analyses were performed to assess the relationship between changes in gait harmonicity (Δ Ф-bonacci gait number = T1 − T0) and performance on both the TUG-DT and the single-task TUG, using Spearman’s rank correlation coefficient.

To quantify global clinical motor changes between T0 and T1, we constructed a composite index termed the Clinical Motor Composite Change Index (CMCCI), based exclusively on clinical scales reflecting Parkinson’s disease severity. Specifically, UPDRS subsection scores I–IV and Hoehn & Yahr (H&Y) stage were included, capturing non-motor experiences of daily living (UPDRS I), motor experiences of daily living (UPDRS II), clinician-rated motor signs (UPDRS III), motor complications (UPDRS IV), and global disease stage (H&Y) (Goetz et al., 2004; Fahn et al., 1987; Hoehn and Yahr, 1967). Functional mobility tests were intentionally excluded to avoid conceptual overlap and statistical circularity with the locomotor outcomes.

For each subject *i* and each included clinical scale *j* (with *j =* 1, …, *k*), a pre–post change score was computed as: $\Delta X_{\mathrm{ij}}=X_{\mathrm{ij}}(T1)-X_{\mathrm{ij}}(T0)$ . Because the included scales have different ranges and variability, change scores were standardized across participants within each scale using a *z*-transformation: $z(\Delta X_{\mathrm{ij}})=((\Delta X_{\mathrm{ij}}-\mu_{\mathrm{\Delta j}})/\sigma_{\mathrm{\Delta j}})$ where $\mu_{\mathrm{\Delta j}}$ and $\sigma_{\mathrm{\Delta j}}$ denote, respectively, the mean and the standard deviation of ∆*X* for the *j*-th scale computed across the study sample.

The CMCCI for subject *i* was then defined as the average of standardized change scores across the *k* clinical scales:


$${\text{CMCCI}}_{i}=\frac{1}{k}\sum_{j=1}^{k}z(\Delta X_{\mathrm{ij}})$$ (6)

Negative CMCCI values indicate overall clinical improvement, positive values suggest worsening, while values close to zero indicate clinical stability. Spearman’s rank correlations were then performed between CMCCI and both the change in gait harmonicity (Δ Ф-bonacci gait number) and TUG-DT (ΔTUG-DT) to assess the relationship between clinical motor improvement and locomotor performance.

Statistical analysis of the data was performed using Matlab R2023b (The MathWorks, Natick, MA, United States) as the computing and programming platform. For the described demographic and clinical data of the sample, frequencies with relative percentage, mean value with standard deviation, and median value were calculated for the categorical, continuous, and ordinal variables, respectively. To minimize confounding effects, all assessments were performed in a standardized ON-medication state, and disease severity was controlled through inclusion criteria (Gualerzi et al., 2024; Hou et al., 2019). Inferential analysis was conducted using the Kolmogorov–Smirnov test to evaluate the normality of the data distributions. Given the non-normal distribution of the data and small sample size, clinical assessment scores and spatiotemporal parameters were compared between baseline (T0) and post-intervention (T1) using the non-parametric Wilcoxon signed-rank test for paired data.

## 3 Results

**Figure 2:**
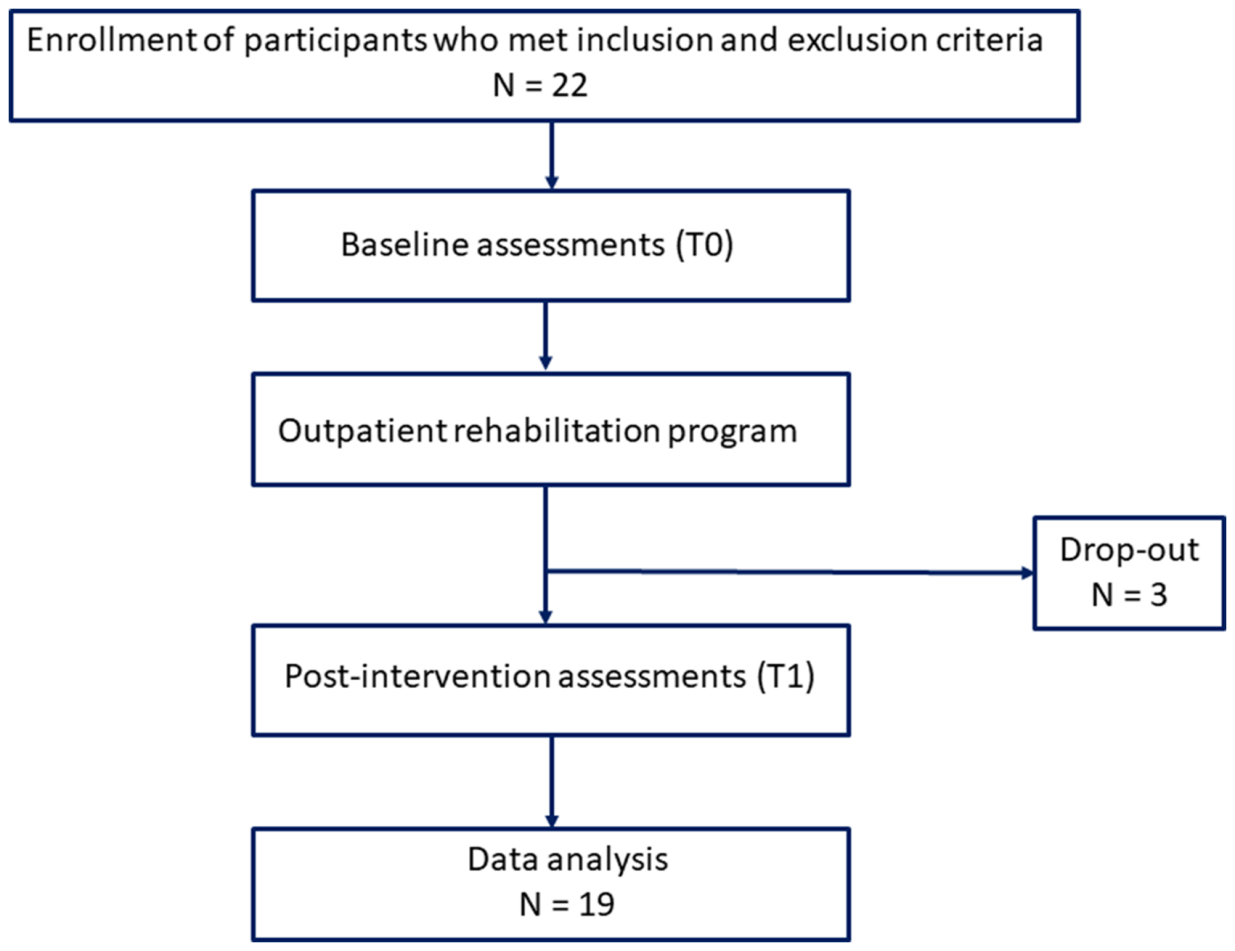
Flowchart of the study.

The study included participants (twenty-two) who met the inclusion and exclusion criteria were recruited (as shown in the Flowchart—Figure 2). All participants underwent clinical and instrumental assessments at T0 (baseline; pre-rehabilitation treatment) and subsequently started their rehabilitation program in a day-hospital setting. At the end of the 30-day rehabilitation period, clinical and instrumental assessments were performed again at T1 (post-rehabilitation treatment). Ultimately, data from the remaining 19 participants were included in the analysis.

Based on the examination of demographic and clinical characteristics (Table 1), the mean age of the participant was 72.2 ± 7.49 years. The duration of the disease (calculated from the year of diagnosis) was 7.0 ± 2.62 years. Clinical assessment showed that the median Hoehn & Yahr stage remained unchanged with a range of 2–2.5. UPDRS scores did not exhibit significant variations following the 30-day rehabilitation intervention, despite an improvement found only in the UPDRS III which involves the motor domain. A slight improvement was observed in the PDQ-8, with a Δ (T1 − T0) of 0.5. MoCA scores remained stable between T0 and T1. A statistically significant improvement (*p* < 0.05) was found in the Mini-BESTest, increasing from a score of 24.5 (range 17–28) at T0 to 27 (range 18–28) at T1. Statistically significant improvements (*p* < 0.05) were also observed in both the TUG and TUG-DT: TUG time decreased from 10.27 ± 3.39 s at T0 to 9.11 ± 3.15 s at T1, while TUG Dual-Task time decreased from 13.23 ± 4.93 s at T0 to 11.1 ± 3.81 s at T1. The 6MWT showed statistically significant improvement (*p* < 0.05), with a Δ (T1 − T0) distance of 59.6 m. The 10mWT time and NFOG-Q score remained essentially unchanged. Similarly, the confidence level measured using the ABC scale did not vary significantly between T0 and T1.

**Table 1:** Demographic and clinical characteristics of the study participants.

Variables	Participants characteristics (*N* = 19)				Normative reference data
	T0	T1	∆ (T1 − T0)	Cohen’s *d* [95% CI]	
Age (years)	72.27 ± 7.49		—	—	68.53 ± 3.56
Gender male, *n* (%)	12 (63.2%)		—	—	9 (60.0%)
Disease onset (years)	7.0 ± 2.62		—	—	—
H&Y	2.5 (2–2.5)	2.5 (2–2.5)	—	—	—
UPDRS I	11 (3–30)	11 (6–30)	—	—	—
UPDRS II	9.5 (4–23)	9 (4–23)	—	—	—
UPDRS III	11 (5–21)	9 (5–18)	—	—	—
UPDRS IV	4 (0–13)	4 (0–13)	—	—	—
UPDRS TOT	38 (15–79)	37 (14–79)	—	—	—
PDQ-8	5.5 (0–16)	6 (1–19)	—	—	—
MoCA	28 (22–29)	28 (22–30)	—	—	—
Mini-BESTest	**24.5 (17–28)**	**27 (18–28)**	—	—	—
TUG (sec)	**10.27 ± 3.39**	**9.11 ± 3.15**	−1.16	−0.35 [−1.00, 0.30]	6.8 ± 2.91
TUG dual-task (sec)	**13.23 ± 4.93**	**11.1 ± 3.81**	−2.13	−0.48 [−1.15, 0.20]	11.0 ± 1.54
6MWT (m)	**394.6 ± 70.11**	**454.2 ± 63.5**	+59.60	0.89 [0.14, 1.64]	500.09 ± 18.33
10mWT (sec)	6.42 ± 0.99	6.40 ± 0.88	−0.02	—	6.1 ± 0.23
NFOG-Q	5 (0–13)	6 (1–13)	—	—	—
ABC (%)	82.19 (19.0–95.6) %	84.70 (43.8–98.1) %	—	—	—

Data are reported as mean ± standard deviation or frequency with percentage (%) or median (min–max). Effect sizes for primary and secondary outcomes are calculated using Cohen’s *d* based on pooled standard deviations [95% CI: Ninety-five percent confidence intervals]. In bold ***p* < 0.05** (Wilcoxon signed-rank test for paired data T0; T1). Normative data from healthy adults are reported as a reference. H&Y, Hoehn & Yahr; UPDRS, Unified Parkinson’s Disease Rating Scale; PDQ-8, Parkinson’s Disease Questionnaire-8 items; MoCA, Montreal Cognitive Assessment; Mini-BESTest, Mini Balance Evaluation Systems Test; TUG, Time Up and Go Test; 6MWT, 6 Minute Walk Test; 10mWT, 10 meters Walk Test; NFOG-Q, New Freezing of Gait Questionnaire; ABC, Activities-Specific Balance Confidence Scale.

Spatiotemporal analysis (Table 2) showed a significant increase in stride length for both limbs, rising from 0.76 ± 0.06 m at T0 to 0.85 ± 0.08 m at T1 for the right lower limb, and from 0.77 ± 0.06 m to 0.89 ± 0.07 m for the left lower limb (*p* < 0.05). Walking average speed also improved significantly, with a ∆ (T1 − T0) of 0.16 ± 0.10 m/s (*p* < 0.05). Cadence increased from 98.43 ± 14.22 steps/min to 100.90 ± 7.23 steps/min, reflecting a statistically meaningful change (*p* < 0.05). Stride length and walking speed are two gait-pattern parameters that show a marked reduction in pwPD during the “ON” state (Chien et al., 2006). Given that cadence is a Levodopa-resistant gait parameter, as demonstrated by Stolze et al. (2001), (Morris et al., 1994) its improvement is of considerable relevance and has a substantial impact on the motor behavior of pwPD.

**Table 2:** Spatiotemporal parameters results.

Spatiotemporal parameters	Spatiotemporal parameters results				Normative reference data	
	T0		T1			
	Right	Left	Right	Left	Right	Left
Stride time (sec)	1.32 ± 0.47	1.36 ± 0.55	1.32 ± 0.24	1.35 ± 0.25	1.10 ± 0.02	1.03 ± 0.02
Stance phase time (sec)	0.84 ± 0.47	0.85 ± 0.56	0.82 ± 0.16	0.83 ± 0.15	0.69 ± 0.06	0.69 ± 0.07
Swing phase time (sec)	0.48 ± 0.07	0.49 ± 0.04	0.46 ± 0.05	0.48 ± 0.08	0.42 ± 0.01	0.42 ± 0.01
Single support time (sec)	0.44 ± 0.02	0.46 ± 0.08	0.45 ± 0.01	0.44 ± 0.01	0.42 ± 0.01	0.41 ± 0.01
Double support time (sec)	0.42 ± 0.51	0.44 ± 0.52	0.40 ± 0.20	0.42 ± 0.50	0.27 ± 0.07	0.27 ± 0.07
Step time (sec)	0.67 ± 0.46	0.65 ± 0.30	0.67 ± 0.10	0.67 ± 0.10	0.56 ± 0.07	0.55 ± 0.07
Stance phase (%)	61.49 ± 4.98	57.94 ± 3.04	61.14 ± 1.53	59.10 ± 2.03	62.02 ± 0.96	62.01 ± 1.23
Swing phase (%)	37.08 ± 3.49	36.56 ± 2.72	38.85 ± 1.53	40.89 ± 2.04	37.99 ± 0.96	38.02 ± 1.26
Single support (%)	35.60 ± 1.37	36.00 ± 2.14	37.26 ± 1.28	36.93 ± 4.80	37.99 ± 1.27	38.02 ± 1.06
Double support (%)	28.73 ± 2.38	28.66 ± 2.79	26.41 ± 2.84	28.35 ± 3.58	23.98 ± 1.52	23.97 ± 1.53
Step time (%)	50.86 ± 2.39	49.14 ± 3.42	49.91 ± 2.80	50.40 ± 4.38	50.01 ± 0.80	50.03 ± 0.86
Stride length (m)	**0.76 ± 0.06**	**0.77 ± 0.06**	**0.85 ± 0.08**	**0.89 ± 0.07**	1.24 ± 0.03	1.04 ± 0.02
Step length (m)	0.40 ± 0.03	0.44 ± 0.03	0.43 ± 0.05	0.45 ± 0.05	0.64 ± 0.02	0.60 ± 0.02
Speed (m/s)	**0.62 ± 0.06**		**0.78 ± 0.08**		1.14 ± 0.04	
Cadence (step/min)	**98.43 ± 14.22**		**100.90 ± 7.23**		109.77 ± 5.15	

Data are reported as mean ± standard deviation. Normative data from healthy adults are reported as a reference. In bold ***p* < 0.05** (Wilcoxon signed-rank test for paired data T0; T1).

Spatiotemporal analysis (Table 3) revealed a significant decrease in the Ф-bonacci gait number from 1.86 ± 0.82 at T0 to 1.50 ± 0.79 at T1 (*p* < 0.05). As this index is derived from A1—Self Similarity, A2—Symmetry, and A3—Double Support (DS) Consistency) (Cocco et al., 2025), it is noteworthy that both A1 and A3 showed significant reductions. Specifically, A1 decreased from 0.58 ± 0.12 to 0.47 ± 0.20, and A3 from 1.04 ± 0.87 to 0.73 ± 0.32 (*p* < 0.05). These results represent significant information on the walking performance in terms of harmonicity (self-similarity A1) and DS consistency (A3). The Φ-bonacci and dual-task improvements showed medium standardized effects, whereas walking endurance (6MWT) demonstrated a large effect size, suggesting meaningful functional adaptation. The results concerning the Φ-bonacci gait number clarify the significance of the study, with the Φ-bonacci gait number representing a key indicator for monitoring the participants’ rehabilitation progress on walking performance.

**Table 3:** Gait harmonicity descriptors.

Gait harmonicity descriptors	Gait harmonicity results				Normative reference data
	T0	T1	∆ (T1 − T0)	Cohen’s *d* [95% CI]	
Ф-bonacci gait number	**1.86 ± 0.82**	**1.50 ± 0.79**	−0.36	−0.45 [−1.10, 0.20]	0.86 ± 0.26
A1	**0.58 ± 0.12**	**0.47 ± 0.20**	−0.11	—	0.03 ± 0.02
A2	0.16 ± 0.11	0.14 ± 0.13	−0.02	—	0.003 ± 0.003
A3	**1.04 ± 0.87**	**0.73 ± 0.32**	−0.31	—	0.21 ± 0.14
GC	1.16 ± 0.09	1.10 ± 0.08	−0.06	—	1.01 ± 0.01

Data are reported as mean ± standard deviation. Effect size for primary outcome is calculated using Cohen’s *d* based on pooled standard deviations [95% CI: Ninety-five percent confidence intervals]. Normative data from healthy adults are reported as a reference. In bold ***p* < 0.05** (Wilcoxon signed-rank test for paired data T0; T1).

**Figure 3:**
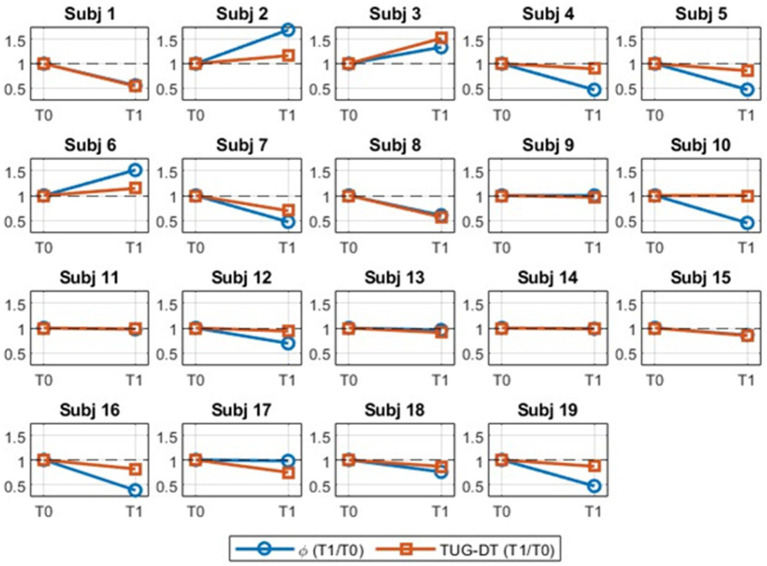
Time course (pre-treatment T0 and post-treatment T1) of the Ф-bonacci gait number (blue) and the TUG-DT (orange) on a normalized scale.

Figure 3 shows the trajectories of the Ф-bonacci gait number (blue) and the TUG-DT score (orange) across T0 and T1 on a normalized scale. Each “Subj” represents an individual participant. The two indices exhibited concordant trends (blue and orange lines) in all 19 individuals. Overall, 84.2% of participants showed either improvement or stability over time (from T0 to T1), specifically, 63.2% of persons showed an improvement in motor performance both in the TUG test under dual-task condition and in terms of gait harmonicity (after 30 days of rehabilitation) and 21.0% remaining stable.

Furthermore, the aligned trends indicate a consistent association between cognitive and motor performance in dual-task conditions, whereby changes in one domain are accompanied by corresponding changes in the other. Even though, participants achieved the same cognitive test scores, which represents cognitive stability, at the same time, clinical results in motor performance tests and dual-task tests showed statistically significant improvements. This pattern is consistent with the known interdependence between motor control and cognitive–motor dual-tasking performance.

**Figure 4:**
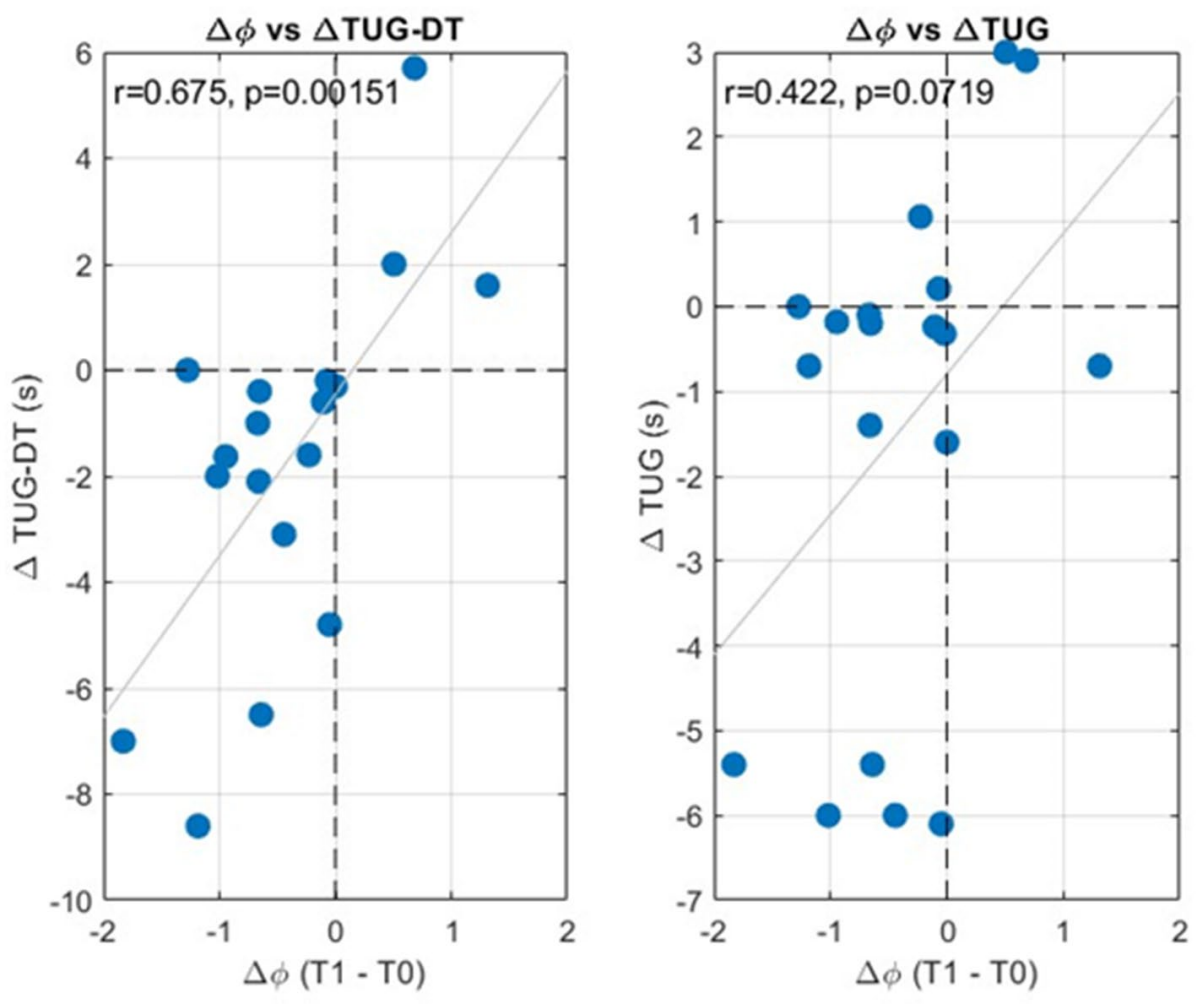
Linear correlation analysis using Spearman’s coefficient (*r*) on the Δ TUG-DT vs. Δ Ф-bonacci gait number and on the Δ TUG vs. Δ Ф-bonacci gait number.

Spearman’s rank correlation analysis revealed also a significant positive association between the CMCCI and the change in gait harmonicity (Δ Ф-bonacci gait number; *r* = 0.640, *p* = 0.013). Given that improvement is reflected by negative values for both CMCCI and ΔФ, the positive correlation (*r* = 0.640) indicates that greater clinical motor improvement was associated with a larger reduction in the Ф-bonacci gait number. To examine the association between the Ф-bonacci gait number and TUG, and between the Ф-bonacci gait number and TUG-DT, Spearman’s rank correlations were computed (Figure 4). The correlation coefficient for Ф-bonacci gait number and TUG-DT revealed a strong relationship between the two variables, with *r* = 0.675 and high statistical significance (*p* < 0.05). Because improvement corresponds to negative deltas for both indices, the positive correlation (*r* = 0.675, *p* < 0.05) indicates that individuals who showed a larger reduction in the Ф-bonacci gait number also exhibited a larger reduction in TUG-DT time. This finding highlights the relevance of the link between the motor domain, captured by the Ф-bonacci gait number, and the cognitive–motor domain, assessed through TUG-DT.

## 4 Discussion

### 4.1 Main findings

The present observational pilot study was designed to explore the longitudinal behavior of the Φ-bonacci gait number, as a composite harmonicity metric, along with its relationship with clinical motor evolution and dual-task mobility in pwPD participating in a conventional multidisciplinary outpatient rehabilitation program (Kalbe et al., 2023; Goffredo et al., 2024; Cocco et al., 2025; Verrelli et al., 2025). Overall, our results show that, despite the short observation window and the neurodegenerative nature of PD, most participants exhibited either improvement or stability in motor performance and gait quality. Specifically, we observed significant gains in functional mobility (TUG, TUG-DT, 6MWT, Mini-BESTest), accompanied by increased stride length, walking speed and cadence, and by a reduction of the Ф-bonacci gait number, mainly driven by changes in self-similarity (A1) and double-support consistency (A3) (Cocco et al., 2025; Verrelli et al., 2025). Importantly, changes in gait harmonicity were significantly associated with both clinical motor improvement, captured by the CMCCI, and dual-task performance (Verrelli et al., 2021; Cocco et al., 2025; Verrelli et al., 2025; Fahn et al., 1987; Pirtošek, 2024).

Significant improvements were observed in the TUG and TUG-DT tests (Figure 3) reflecting a reduction in core motor symptoms, particularly bradykinesia and difficulties in postural transitions. The reduction in TUG execution time indicates an improved ability to initiate gait, perform turning movements, and manage sit-to-stand and stand-to-sit transitions, which are essential components of independence in daily activities. These functional gains are directly associated with a reduced risk of falls, as suggested by the well-established relationship between TUG performance and social participation in pwPD (Armstrong and Okun, 2020). The more pronounced improvement observed simultaneously in the TUG-DT and in the Ф-bonacci gait number (Figure 3) highlights a specific interaction between motor and cognitive functions. This finding suggests an enhanced allocation of attentional resources during walking, a function frequently impaired in Parkinson’s disease and critically involved in real-life situations. The literature consistently shows that difficulties encountered during dual-tasking are closely associated with increased fall risk, reduced participation, and decreased quality of life in this population. In parallel, the significant increase in the distance covered during the 6MWT (Table 1) reflects an improvement in walking endurance and overall functional capacity. Improvements in spatiotemporal gait parameters (Table 2), particularly increased stride length, walking speed, and cadence, indicate a reduction in gait hypokinesia and a better organization of motor output. Notably, the increase in cadence classically described as relatively insensitive to dopaminergic therapy supports the hypothesis of a specific effect of motor training and gait re-education, independent of pharmacological treatment (Morris et al., 1994; Marino et al., 2023). Finally, the significant reduction in the Ф-bonacci gait number following rehabilitation (Figure 3) indicates an improvement in overall gait harmonicity. From a clinical perspective, these changes reflect a smoother, more stable, and better-coordinated walking pattern, with reduced gait variability and irregularity associated with decreased instability and improved locomotor safety in pwPD (Iosa et al., 2016; Verrelli et al., 2021); however, despite the observed statistical significance, their clinical relevance should be interpreted with caution given the pilot nature of the study.

In addition to conventional spatiotemporal parameters, the Ф-bonacci gait number provided a compact description of the global organization of gait, capturing the joint evolution of self-similarity, symmetry, and double-support coherence following rehabilitation. The significant reduction in the Ф-bonacci value from T0 (1.86 ± 0.82) to T1 (1.50 ± 0.79) indicates a shift toward a more harmonious, stable, and temporally coherent gait pattern. At the component level, the most pronounced changes were observed in A1 (T0: 0.58 ± 0.12; T1: 0.47 ± 0.20) and A3 (T0: 1.04 ± 0.87; T1: 0.73 ± 0.32) reflecting, respectively, increased stride-cycle self-similarity and greater consistency of the double-support phases. In contrast, step symmetry—A2 (T0: 0.16 ± 0.1; T1: 0.14 ± 0.13) exhibited more limited modulation, suggesting that rehabilitation predominantly influenced the internal temporal structure of gait and inter-limb transitions rather than pure left–right spatial balance. These findings extend our previous cross-sectional work (Cocco et al., 2025), in which the Ф-bonacci gait value was shown to discriminate between pwPD and healthy controls and to correlate with clinical measures, demonstrating that the same index is also sensitive to short-term changes, as a longitudinal marker, potentially complementary to more traditional gait parameters.

### 4.2 Motor–cognitive interaction in dual-task performance and clinical implications

The results of the present study confirm the existence of an interaction between motor and cognitive capacities in Parkinson’s disease. After one month of conventional rehabilitation, a significant improvement in performance on the TUG-DT was observed (Figure 3), while global cognitive scores, assessed using the MoCA, remained stable (Table 1). The literature describes dual-task walking as a sensitive marker of underlying cognitive deficits in PD, particularly impairments in attentional and executive functions, which substantially contribute to postural instability and increased risk of falls. Several studies have demonstrated that cognitive decline is associated with a greater dual-task cost, increased gait variability, and reduced walking efficiency, thereby limiting the effectiveness of motor rehabilitation interventions (Alcock et al., 2016). In this context, the absence of cognitive deterioration observed in our cohort appears to represent a favorable condition that enabled the expression of motor benefits induced by rehabilitation. The significant association between the reduction in the Ф-bonacci gait number and the improvement in TUG-DT performance (Figure 4) further supports this interpretation. Previous studies have shown that degradation of gait harmonicity and regularity in pwPD is exacerbated under dual-task conditions, reflecting a disruption of motor–cognitive coupling (Iosa et al., 2016; Verrelli et al., 2021). The decrease in the Ф-bonacci gait number observed after rehabilitation suggests a partial restoration of this coupling, allowing the locomotor system to maintain a more stable and coherent temporal organization despite an increased cognitive load.

These findings are also consistent with neurophysiological models proposed in the literature, according to which gait control in Parkinson’s disease relies heavily on cognitive resources due to impaired automatic motor control mechanisms. When cognitive functions remain stable, subjects are able to partially compensate for motor deficits through enhanced attentional control, thereby facilitating adaptation to environmental constraints and complex walking situations (Armstrong and Okun, 2020; Kalbe et al., 2023). Furthermore, our findings suggest that the motor–cognitive relationship in Parkinson’s disease may be better interpreted as an interdependence, in which preserved cognitive resources enable motor adaptation, while changes in motor organization actively shape motor–cognitive performance under dual-task conditions (Smeeton et al., 2021).

First, the use of indicators such as the Ф-bonacci gait number represents an additional objective tool for clinicians, enabling the assessment of locomotor performance with a level of sensitivity that is difficult to achieve using clinical scales alone. Indeed, instrumented gait parameters derived from inertial sensors provide added diagnostic and follow-up value by allowing the detection of subtle alterations in the organization of locomotion. Moreover, recent literature highlights that the integration of instrumented measures facilitates precise longitudinal monitoring and improves the understanding of patients’ responses to therapeutic interventions (Del Din et al., 2016; Petzinger et al., 2013). This approach is particularly relevant in the context of a progressive disorder such as PD.

Next, our results confirm that the cognitive load associated with walking influences motor performance in pwPD. This interaction highlights that therapeutic management cannot be limited to an exclusively motor approach but should instead be oriented toward combined motor–cognitive interventions, in order to reflect the complex demands of daily life (Del Din et al., 2016; Petzinger et al., 2013). Furthermore, these observations support the idea that cognitive assessment should not be restricted to neuropsychological tests conducted in decontextualized conditions, but should incorporate ecologically valid functional assessments, such as dual-task walking paradigms. Such assessments allow the identification of attentional and executive impairments, as confirmed by recent literature (Rochester et al., 2014).

Finally, another important aspect concerns fall prevention, which represents a major issue in pwPD. Indeed, the literature shows that combined motor and cognitive deficits are associated with a significant increase in fall risk (Lord et al., 2013). Consistently, recent studies have shown that Ф-bonacci–based gait metrics derived from wearable sensors can objectively capture residual locomotor instability and sensory reweighting deficits in persons with vestibular disorders, supporting their role as clinically meaningful digital biomarkers beyond PD (Francavilla et al., 2026).

The integration of instrumented gait measures and dual-task training programs within rehabilitation interventions may therefore help to reduce this risk and to strengthen functional autonomy, with a positive impact on quality of life.

### 4.3 Limitations

This study presents limitations that should be considered when interpreting the results.

First, the assessment of cognitive functions relied on the use of the MoCA, which is a widely used global screening tool in clinical practice. Although the MoCA allows for an initial estimation of overall cognitive functioning, it does not provide a detailed analysis of specific cognitive domains, such as executive functions, divided attention, or information processing speed, which play a central role in dual-task situations. This aspect can limit the ability to explore in depth the cognitive mechanisms underlying the observed locomotor adaptations.

Remark: although normative reference data have been provided to exhaustively have a comparison between healthy and pathological individuals, there is no further control group of subjects with the same pathology as the experimental group who are treated in parallel, in line with the aim of the study. In fact, it was not designed as a clinical efficacy study but as a longitudinal observational analysis aimed at evaluating the sensitivity of a new gait index.

### 4.4 Future directions

The relatively small sample size should be taken into account when interpreting the results of this study. While the observed findings were consistent and statistically significant for several parameters, future investigations including larger samples may help to further enhance statistical power, support the generalizability of the results, and allow a more in-depth exploration of interindividual variability in response to rehabilitation.

Based on the data collected to date, future studies may consider the design of a randomized controlled trial, including a control group with comparable clinical and demographic characteristics, to further explore the motor–cognitive interplay, potentially through the implementation of rehabilitation approaches specifically targeting the cognitive domain.

## 5 Conclusion

This observational pilot study examined the evolution of gait harmonicity and dual-task mobility over a one-month multidisciplinary outpatient rehabilitation program in pwPD. In parallel with conventional spatiotemporal improvements, it was observed a significant reduction in the Φ-bonacci gait number, mainly attributable to changes in stride self-similarity and double-support consistency. These modifications were accompanied by improvements in dual-task performance and were significantly associated with composite clinical motor change, as quantified by the CMCCI. Within this framework, the Φ-bonacci gait number demonstrated responsiveness to longitudinal modifications in gait temporal organization and showed coherent associations with both motor and motor–cognitive measures. The current findings support the feasibility of using harmonicity-based gait metrics as complementary tools for longitudinal monitoring in Parkinson’s disease. Future controlled studies with larger samples and domain-specific cognitive assessments are needed to further clarify the clinical utility and mechanistic implications of the Φ-bonacci gait number in rehabilitation settings.

## Data Availability

The raw data supporting the conclusions of this article will be made available by the authors, without undue reservation.
